# Relationships and Sexuality in Patients with Inflammatory Bowel Disease: Experiences of Patients and Healthcare Providers in Sweden

**DOI:** 10.3390/jcm14217608

**Published:** 2025-10-27

**Authors:** Emma Druvefors, Pär Myrelid, Erik Florwald, Anette Forsell, Francesca Bello, Sven Almer, Susanna Jäghult

**Affiliations:** 1 Department of Biomedical and Clinical Sciences, Faculty of Health Sciences, Linköping University, SE-58183 Linköping, Sweden; par.myrelid@liu.se; 2 Department of Surgery, County Hospital Ryhov, SE-55185 Jönköping, Sweden; 3 Department of Surgery, Linköping University Hospital, SE-58185 Jönköping, Sweden; 4 Department of Medicine, Huddinge (MedH), Karolinska Institutet, SE-14183 Stockholm, Sweden; erik.florwald@ki.se; 5 Department of Medicine, Ersta Hospital, SE-11691 Stockholm, Sweden; anette.forsell@erstadiakoni.se; 6 Center for Digestive Health, Department of Gastroenterology, Dermatology, Rheumatology Karolinska University Hospital, SE-17164 Stockholm, Sweden; francesca.bello@regionstockholm.se; 7 Department of Medicine Solna, Karolinska Institutet, SE-17176 Stockholm, Sweden; sven.almer@ki.se; 8 Department of Clinical Science and Education, Karolinska Institutet, Södersjukhuset, SE-11883 Stockholm, Sweden; susanna.jaghult@gmail.com

**Keywords:** inflammatory bowel disease, relationships, sexuality

## Abstract

**Background/Objectives**: The aim of this study was to investigate the experiences of Swedish patients with inflammatory bowel disease (IBD) regarding intimacy and sexuality-related issues, and to explore both patients’ and healthcare professionals’ perspectives on discussing these topics. **Methods**: This cross-sectional cohort study used two internet-based questionnaires: one targeting patients and the other healthcare professionals. The patient survey examined the impact of IBD and its treatment on relationships and sexuality, as well as expectations on healthcare support. The survey of healthcare professionals focused on experiences of discussing sexuality-related topics with IBD patients. Responses were analyzed using both quantitative and content analysis. **Results**: A total of 556 IBD patients and 118 healthcare professionals responded. Among patients, 78% reported difficulties related to relationships and sexuality, with physical symptoms like pain, fecal urgency, and bloating, and psychological problems such as fear of leakage and reduced sexual desire. Over half wished for these issues to be addressed in routine care, yet 84% had never initiated such discussions themselves. Among healthcare professionals, 23% never addressed issues of relationship and sexuality with patients, and another 50% did so only occasionally. Only 15% had access to qualified sexologists for referrals, and just 8% offered sexual rehabilitation after pelvic surgery. **Conclusions**: Sexual health is frequently compromised in IBD patients, especially in women, but remains insufficiently addressed in clinical practice. Both patients and healthcare professionals expressed a need for more open discussions about relationships and sexuality. Improving care requires routine screening, multidisciplinary support, and the development of guidelines for managing sexual dysfunction in IBD.

## 1. Introduction

Inflammatory bowel disease (IBD) is a heterogeneous inflammatory condition characterized by chronic inflammation of the bowel. The relapsing intestinal inflammation frequently results in abdominal pain, fatigue, weight loss, and diarrhea [1]. The most common IBD subtypes are ulcerative colitis (UC) and Crohn’s disease (CD). IBD typically presents during adolescence or early adulthood [2]. Accordingly, the disease onset coincides with the time many people establish relationships [3]. Intimacy, sexuality, and reproductive issues are, for this reason, of great importance to patients with IBD [4], and they seek information and support often without receiving it [5,6].

Sexual dysfunction (SD) is defined as “a sexual problem that is persistent or recurring and causes marked personal distress or interpersonal difficulty” [7]. SD and reduced fertility are seen in both female and male IBD patients [3,8,9,10,11,12,13]. By nature, these concerns differ between women and men, as well as depending on the subtype of IBD [13,14]. Disease-specific factors such as disease duration and activity, drug use, and surgical procedures may contribute to the clinical picture, and control of IBD activity does not appear to be sufficient to improve patients’ quality of life [15]. Overall, there is a lack of knowledge in how to best support patients with IBD within the areas of intimacy and sexuality [3,12]. Clinical guidelines for the treatment of SD in patients with IBD are lacking [15], although patients inquire about information from an IBD specialist. Improved clinical awareness and understanding of the etiology, risk factors, and impact of SD for patients with IBD are suggested to result in improved diagnosis, care, and ultimately better health and wellbeing for this patient population [3,12,16].

The aim of this study was to investigate the experiences of intimacy and sexuality related problems among Swedish patients with IBD and to explore patients’ and healthcare professionals’ experiences of discussing these topics.

## 2. Materials and Methods

### 2.1. Study Design

This study was a cross-sectional cohort investigation that utilized two distinct, internet-based, and study-specific questionnaires (Supplementary files S1 and S2). The first questionnaire was designed to examine the experiences of patients with IBD regarding how the disease and its treatment impact relationships and sexuality, as well as how these issues are taken care of by the healthcare system. It included seven questions, four of which were general (age, gender, type of disease, and previous surgeries). The remaining questions had a qualitative approach with free-text answers, concerning three main areas: (1) challenges related to intimate relationships and sexuality experienced as a consequence of IBD, (2) the type of support or assistance patients would like to receive from healthcare, and (3) whether issues concerning relationships and sexuality have been raised in healthcare encounters.

The second questionnaire explored healthcare professionals’ perspectives on discussing relationship and sexuality-related matters with IBD patients. It contained nineteen questions, six of which were general (profession, gender, age, and work experience). The remaining questions focused on patients’ main concerns (by gender), the support and referrals offered, how and when sexuality is discussed in clinical practice, providers’ access to specialist resources, their training in sexual and reproductive health, their ability to respond to patient questions, and the availability of sexual rehabilitation after pelvic surgery.

Both surveys were presented to representatives of the Swedish Patient Association for diseases of the gastrointestinal tract (Magtarmförbundet) and healthcare providers, respectively, and underwent several revisions before reaching their final forms. The online patient survey was closed after eight weeks due to the large number of responses. The healthcare professionals’ survey remained open for five months. This study was approved by the national ethical review board, Etikprövningsmyndigheten (registration number: 2025-00089-01).

### 2.2. Study Population

From the Swedish Patient Association, all members with IBD were identified. Healthcare professionals were identified through professional associations working with IBD patients: the Swedish Society of Gastroenterology, the Swedish Society for Colon and Rectal Surgeons, the Swedish Association for Gastroenterological Nursing, the Swedish Society for Pediatric Gastroenterology, Hepatology and Nutrition, the Swedish Society of Young Gastroenterologists, and the Association for Stoma Therapists and Nurses in Colorectal Care, as well as users of the Swedish Inflammatory Bowel Disease Registry.

### 2.3. Survey Distribution

For the patient survey, an invitation letter was sent to all members of the Swedish Patient Association for diseases of the gastrointestinal tract with a registered IBD diagnosis, and for the healthcare professionals survey, an invitation letter was sent to the professional societies described above. The letters contained information about the study and a link to the electronic questionnaire hosted on the secure Webropol^®^ research environment (Helsinki, Finland). All answers were anonymous and could not be traced back to individual responders. One reminder was sent halfway through the survey’s opening time. Participants were not offered any kind of economic or other compensation.

### 2.4. Statistical Analysis

For the patient survey, characteristics, including age, gender, diagnosis, and previous surgical procedures, were collected for all respondents. Descriptive statistics were reported as means, medians, or proportions, as appropriate. Comparisons between groups were made using χ^2^ statistics. Free-text responses were analyzed qualitatively using a manual content analysis and a frequency analysis approach. This method was used to examine and quantify how often specific words, phrases, or themes occurred within the dataset. By identifying the frequency of particular terms or topics, dominant trends and key topics can be discerned, providing an understanding of the primary concerns, perceptions, and priorities related to the topic. The manual content analysis was performed in Microsoft Excel^®^ for Microsoft 365, version 2024 (Microsoft Corporation, Redmond, WA, USA) by one researcher (SJ). The occurrence of specific words, phrases, and topics within the dataset was systematically quantified, and the findings were subsequently reviewed and discussed with another researcher (SA) to ensure consistency and analytical rigor.

For the healthcare professional survey, characteristics, including profession, age, gender, and number of working years in the IBD field, were gathered for all respondents. Characteristics and responses concerning relationships and sexuality were reported as means, medians, or proportions, as appropriate. The analyses were conducted in SPSS version 29.0.2.0.

## 3. Results

From the Swedish Patient Association, 2274 patients with an IBD diagnosis were identified. The survey was completed by 556 individuals, giving a response rate of 24%. The total number of healthcare professionals who received the request to fill in the second survey through professional associations was not recorded, but 118 responded to the questionnaire.

### 3.1. The IBD Cohort

The patients, on average, were 55 years old at the time of this study (Table 1), and the majority (69%) were women. The distribution between UC (46%) and CD (45%) was even, while microscopic colitis (6%) and IBD unclassified (3%) were uncommon. About a quarter (26%) had previously undergone surgery due to IBD.

Most responders (78%) reported problems related to relationships and sexuality (Table 2). Common physical issues included pain (*n* = 76), fecal incontinence or urgency (*n* = 61), and bloating or gas (*n* = 48). Frequent psychological problems were fear of fecal leaking (*n* = 72), decreased sexual desire (*n* = 62), and fatigue (*n* = 42). Several patients (*n* = 12) reported the need to carefully plan sexual activity, whereas others (*n* = 13) indicated they avoided intimate relationships altogether.

Women reported greater difficulties regarding relationships and sexuality than men, with no differences between diagnoses (Table 3) or between patients who had undergone surgery and those who had not.

Comparative analyses between men and women for the six most frequently reported problems revealed that women experienced significantly greater symptom severity across most domains. Specifically, women reported more problems related to pain (92% vs. 8%, *p* < 0.001), worries about leakage (82% vs. 18%, *p* < 0.001), decreased sexual drive (80% vs. 20%, *p* < 0.001), loss of energy (83% vs. 17%, *p* < 0.001), and gas/bloating (95% vs. 5%, *p* < 0.001). No significant sex differences were observed for fecal incontinence, leakage, or urgency (48% vs. 52%, NS) (Table 3).

A majority (64%) of respondents considered healthcare professionals to be essential in addressing sexuality and related concerns as a natural part of care. Despite this, 84% of participants had never initiated discussions on these topics themselves, attributing this to factors such as embarrassment, time constraints during outpatient visits, or limited overall interaction with healthcare providers. In addition, many sought access to a psychologist or social worker, ideally with involvement from their partner.

### 3.2. The Healthcare Professional Cohort

Among the healthcare respondents, 54% were IBD nurses, 29% were gastroenterologists, 10% were surgeons, and 7% held other professions; no dietitians participated (Table 4). Most were female (76%), 22% were male, and 2% did not specify their gender. The majority worked at university hospitals (51%), followed by county hospitals (15%), private hospitals (14%), district hospitals (13%), private healthcare receptions (6%), and primary care (1%). Median IBD experience was 13 years (IQR 15), and median age was 47 years (IQR 13).

**Figure 1:**
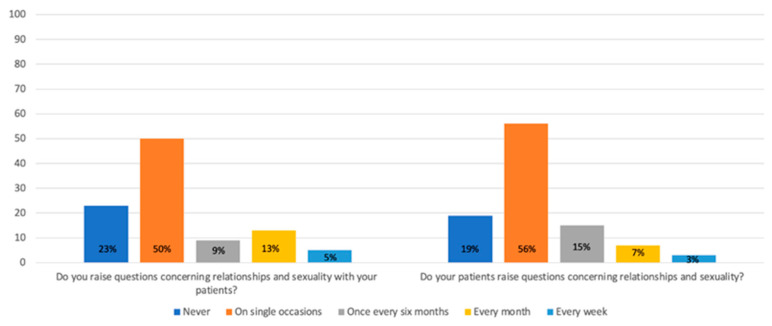
Results from the healthcare professional survey concerning discussions with patients with inflammatory bowel disease regarding relationships and sexuality.

**Figure 2:**
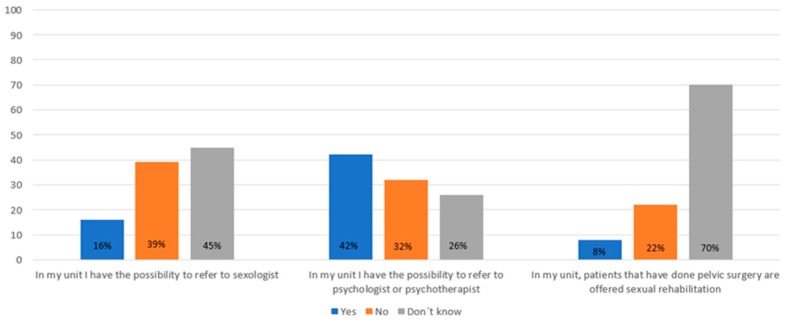
Results from the healthcare professional survey concerning the possibility of referring to other specialists within the area of relationships and sexuality.

Overall, responses were consistent across professional groups; therefore, results are presented for the entire cohort. Half of the respondents indicated that they occasionally address issues related to relationships and sexuality with their patients, while 23% reported that they never discuss these topics (Figure 1). Similarly, 56% stated that patients occasionally initiate such conversations, whereas 19% said this never happens (Figure 1). Only 16% of respondents reported having access to a sexologist, while 42% could refer patients to a psychologist or psychotherapist (Figure 2). Only 8% of the healthcare providers reported that they offered sexual rehabilitation to patients following pelvic surgery.

When patients with IBD raise concerns related to relationships and sexuality, the most frequently reported issues, according to healthcare professionals, include fertility concerns among women and erectile dysfunction among men. Healthcare professionals also observed that both male and female patients experience reduced sexual desire, feelings of unattractiveness, and anxiety about possible fecal leakage. Issues and concerns related to the presence of a stoma were also frequently mentioned. Among women, pain during intercourse—often associated with perianal fistulas or abdominal discomfort—was commonly reported.

## 4. Discussion

This study highlights the often underrecognized impact of IBD on interpersonal relationships and sexual health, revealing substantial gaps between patients’ expectations and how these issues are addressed within clinical settings. The majority of patients with IBD responding to the survey reported significant challenges related to relationships and sexuality, with physical symptoms like pain, fecal urgency, and bloating, as well as psychological factors such as fear of leakage and reduced sexual desire, all playing key roles in the development of SD. While no differences were found between patients with UC and CD, women reported significantly greater inconvenience in key areas, consistent with Pires et al., who observed higher rates of sexual dysfunction in women with IBD, underscoring the importance of gender-specific assessment and care [17]. Despite the clear need, healthcare professionals do not routinely address these concerns, with only 50% occasionally raising the topic with patients. As a result, individual patients are likely to be seldom, if ever, asked about SD, in line with previous studies [6,18,19].

Our findings align with previous reports that demonstrate how IBD-related symptoms, such as perianal disease or abdominal discomfort, contribute to decreased sexual function and quality of life for both male and female patients [12,14,16,20]. Notably, our results indicate that issues such as erectile dysfunction in men and pain during intercourse in women, often linked to perianal fistulas or pelvic discomfort, are common. Furthermore, psychological factors like feelings of unattractiveness and fear of fecal incontinence, frequently reported by all genders, have a profound impact on intimacy and self-esteem. The presence of a stoma [21] was additionally reported to be associated with SD. These results emphasize the need for a more personalized and comprehensive care that addresses both the physical and psychosocial aspects of IBD [5,14,16,18].

Despite a clear wish (64%) from patients for healthcare providers to initiate discussions about sexuality as a routine part of care, 84% of patients had never themselves brought up the topic. This gap indicates a potential discomfort or reluctance on the part of both patients and healthcare providers in addressing sensitive subjects. Time constraints, perceived embarrassment, and the lack of structured guidelines for discussing sexual health in the context of IBD may contribute to this oversight [5,18]. Furthermore, educational and organizational factors, including underlying unreflected values, inadequate professional training, and limited institutional support, are likely to maintain this discrepancy. This aligns with our previous studies, indicating that healthcare professionals also tend to overlook the closely related topic of fertility [22]. Taken together, the findings suggest a broader pattern of unmet needs in which sensitive, yet crucial, aspects of patients’ sexual and reproductive health are insufficiently integrated into routine care [5,18].

In terms of clinical practice, the availability of resources for managing relational and sexual health concerns remains limited. Although 45% of healthcare professionals reported access to psychological support services, only 15% had the ability to refer patients to a sexologist. This limited access to multidisciplinary care highlights the need for a greater integration of sexual health and psychosocial services within IBD care, given the profound impact of SD on overall patient wellbeing [6,14,18].

The strengths of this study lie in the inclusion of both patient and healthcare professional perspectives, providing a dual lens through which to assess the management of sexual health in IBD. An additional strength is the qualitative approach in the patient survey, enabling patients to freely describe their concerns in their own words. The relatively low patient response rate (24%) introduces a potential selection bias, and we cannot exclude that individuals with more pronounced issues may have been over- or underrepresented. Given the qualitative design, further data collection was deemed methodologically unfeasible, and the survey was closed after exceeding 500 responses. Moreover, the lack of the total number of invited healthcare professionals limits the ability to assess the representativeness of the responses.

## 5. Conclusions

In conclusion, our findings highlight the significant, yet often neglected, impact on quality of life of sexual health issues among patients with IBD, especially women, underscoring the need for routine screening and proactive discussions in clinical practice. Given the complexity of SD in IBD, a multidisciplinary approach, involving gastroenterologists, nurses, psychologists, and sexologists, is critical to addressing the full spectrum of patient needs. Nonetheless, the implementation of such models must take into account potential challenges, including constrained resources, organizational limitations, and deficiencies in professional training. Future research should focus on developing standardized protocols for integrating relational and sexual health discussions into routine IBD care and exploring the benefits of specialized interventions aimed at improving sexual function and quality of life for patients.

## Figures and Tables

**Table 1:** Demographics of the IBD patients (*n* = 556) who responded to the survey.

**Sex, %**	
Women	69
Men	31
Non-binary	0
**Diagnosis, %**	
UC	46
CD	45
MC	6
IBD-U	3
**Previous surgery due to IBD, %**	26
**Age, median (IQR)**	55 (27)

**Table 2:** Problems concerning relationships and sexuality were reported by 556 patients with IBD. Items obtained from qualitative content analysis of free-text responses (numbers in parentheses represent the number of patients). * number of participants reporting the issue.

Physical Problems	Psychological Problems
Pain (bowel, vaginal, fistula, rectum) (76) *	Worries about rectal leakage (72)
Fecal incontinence symptoms (61)	Decreased sexual drive (62)
Gas/bloated (48)	Loss of energy (42)
Flares giving symptoms (21)	Feeling non-attractive (26)
Erection problems/impotence (12)	Worries about the stoma, embarrassment, changed self-image, and impact on self-confidence (17)
Practical problems with stoma (10)	Concern about smelling bad/feeling unhygienic (16)
Fistulas (leakage, seton) (8)	Lack of self-confidence (8)
Treatment administered in the rectum (2)	Shame and general anxiety (4)
Frequent infections in the lower abdomen (1)	

**Table 3:** Relationship and sexuality problems by sex and diagnosis.

Symptom/Problem	Women (%)	Men (%)	CD (%)	UC (%)	*p*-Value (Women vs. Men)	*p*-Value (CD vs. UC)
Overall problems	75	25	47	52	<0.001	NS
Pain (bowel, vaginal, fistula, rectum)	92	8	–	–	<0.001	–
Worries about leakage	82	18	–	–	<0.001	–
Decreased sexual drive	80	20	–	–	<0.001	–
Fecal incontinence/ leakage/urgency	48	52	–	–	NS	–
Loss of energy	83	17	–	–	<0.001	–
Gas/bloating	95	5	–	–	<0.001	–

**Table 4:** Demographics of IBD healthcare professionals (*n* = 118) who responded to the survey.

**Profession, %**	
Gastroenterologists	29
Surgeons	10
IBD nurses	54
Others	7
**Sex, %**	
Women	76
Men	22
Do not want to specify	2
**Workplace, %**	
University hospital	51
County hospital	15
Private hospital	14
District hospital	13
Private healthcare reception	6
Primary care	1
**Number of years working with IBD, median (IQR)**	13 (15)
**Age, median (IQR)**	47 (13)

## Data Availability

Compiled de-identified survey responses will be shown upon request.

## Supplementary Materials

The following supporting information can be downloaded at https://www.mdpi.com/article/10.3390/jcm14217608/s1, Supplementary file S1: Questions to patients; Supplementary file S2: Questionnaire to the healthcare professionals.

## Abbreviations

The following abbreviations are used in this manuscript:

CD	Crohn’s disease
IBD	Inflammatory bowel disease
SD	Sexual dysfunction
UC	Ulcerative colitis
