# Supplementary material for: Relationships and Sexuality in Patients with Inflammatory Bowel Disease: Experiences of Patients and Healthcare Providers in Sweden
Source: J Clin Med. 2025 Oct 27;14(21):7608. doi: 10.3390/jcm14217608 (PMC12610819; doi:10.3390/jcm14217608)
Supplement: Supplementary file 1 [file jcm-14-07608-s001.zip › Supplementary file 1.pdf]

Relationships and sexuality in IBD
Questions to patients

1. Gender

- ◯ Man
- ◯ Woman
- ◯ Non-binary
- ◯ Other
- ◯ Don’t want to specify

2. Age

3. Diagnosis

- ◯ Ulcerative colitis
- ◯ Crohn`s disease
- ◯ Microscopic colitis
- ◯ Lymfocythic colitis
- ◯ Collagenous colitis
- ◯ IBD Unklassified

4. Have you had surgery due to your IBD?

- ◯ Yes
- ◯ No

5. What problems have you encountered concerning relationships and sexuality due to your IBD?

6. What kind of help from the healthcare would you like?

7. Have you raised issues concerning relationships and sexuality in contact with the healthcare?
