# Supplementary material for: Relationships and Sexuality in Patients with Inflammatory Bowel Disease: Experiences of Patients and Healthcare Providers in Sweden
Source: J Clin Med. 2025 Oct 27;14(21):7608. doi: 10.3390/jcm14217608 (PMC12610819; doi:10.3390/jcm14217608)
Supplement: Supplementary file 1 [file jcm-14-07608-s001.zip › Supplementary file 2.pdf]

Relationships and sexuality in IBD
Questions to the healthcare professionals

1. Profession

- ◯ Gastroenterologist
- ◯ Surgeon
- ◯ Reg Nurse
- ◯ Dietician
- ◯ Other

2. Number of years you have been working with IBD

3. Sex

- ◯ Woman
- ◯ Man
- ◯ Non-binary
- ◯ Do not want to specify

4. Age

5. Which region do you work in?

6. My main workplace is at

- County hospital
- Central hospital
- University hospital
- Private healthcare unit
- Primary care
- Private hospital

7. What are the main problems regarding their IBD that your female patients raise concerning relationships and sexuality?

8. What are the main problems regarding their IBD that your male patients raise concerning relationships and sexuality?

9. What kind of help and support have you been able to offer?

- ○ None
- ○ Own councellling
- ○ Medical treatment
- ○ Book additional visits
- ○ Referral to sexologist, psychologist, social worker or other

10. Do you raise questions concerning relationships and sexuality with your patients?

- ○ Never
- ○ On single occassions
- ○ Once every six months
- ○ Every month
- ○ Every week

11. If you ticked question 10 in any of the options that you raise questions concerning intimacy, in what context does this occur?

- ☐ At disease onset
- ☐ On new visits
- ☐ In connection with transition to adult care
- ☐ Before biological treatment
- ☐ Before surgery
- ☐ Other situations

12. Do your patients raise questions concerning relationships and sexuality?

- ○ Never
- ○ On single occassions
- ○ Once every six months
- ○ Every month
- ○ Every week

13. If you ticked question 12 in any of the options that you raise questions concerning intimacy, in what context does this occur?

- ☐ At disease onset
- ☐ On new visits
- ☐ In connection with transition to adult care
- ☐ Before biological treatment
- ☐ Before surgery
- ☐ Other situations

14. In my unit I have the possibility to refer to a sexologist

- ○ Yes
- ○ No
- ○ Don’t know

15. In my unit I have the possibility to refer to a psychotherapist

- ○ Yes
- ○ No
- ○ Don’t know

16. Have you participated in any educations or congresses concerning sexual and reproductive health?

- ○ Never
- ○ Yes, at 1 occassion
- ○ Yes, at 1-3 occassions
- ○ Yes, at 3-6 occassions
- ○ Yes, at more than 7 occassions

17. How many questions from the patients concerning intimacy can you answer yourself

I don't answer any questions myself	0 — slider (0) — 10	I can answer all questions myself

18. Do patients that have undergone pelvic surgery any sexual rehabilitation?

- ○ Yes
- ○ No
- ○ Don't know

19. If you have answered yes in question 18, what kind of rehabilitation?

___
___
___
___
___
